# Effective Factors on the Rate of Growth Failure in Children below Two Years of Age: A Recurrent Events Model

**Published:** 2018-03

**Authors:** Farzad EBRAHIMZADEH, Ebrahim HAJIZADEH, Ahmad Reza BAGHESTANI, Mohammad Reza NAZER

**Affiliations:** 1. Dept. of Biostatistics, School of Medical Sciences, Tarbiat Modares University, Tehran, Iran; 2. Dept. of Biostatistics, School of Paramedical Sciences, Shahid Beheshti University of Medical Sciences, Tehran, Iran; 3. Dept. of Infectious Diseases, Hepatitis Research Center, Lorestan University of Medical Sciences, Khorramabad, Iran

**Keywords:** Growth failure, Children, Survival analysis, Recurrent events model, Proportional rate model

## Abstract

**Background:**

Growth failure, constituting one the health problems in children below 2 yr of age, can lead to major complications such as death or mental, emotional and physical disabilities. The present study aimed to investigate effective factors on growth failure in the height and weight of less than 2 yr old children of Khorramabad, Iran in 2013.

**Methods:**

This present longitudinal retrospective study used stratified and clustered sampling. Based on growth curves in family records, the incidence times of growth failure in height and weight of each child were recorded. In the next stage, using recurrent events model (proportional rate model), along with SAS software (version 9.2), the data were modeled.

**Results:**

According to proportional rate model, the effect of mothers’ educational level on the rate of growth failure in the height and weight of children was significant (*P*=0.046, *P*=0.049) and the effect of fathers’ job was significant only on growth failure in children’s weight (*P*<0.001). However, the effect of other variables, including gender, birth order and exclusive breastfeeding status on children’s growth failure rate was not significant.

**Conclusion:**

Enhancing mother’s awareness in low-income families, in tandem with changing educated mothers’ attitude towards the required skills and guiding principles for feeding children below 2 yr of age, can be conceived of as the most important approach in dealing with growth failure of children.

## Introduction

Growth failure in children indicates whether a child’s height and weight growth, compared to healthy children, is retarded. However, it is not construed as an illness; rather it is a common sign or symptom among several different disorders (1–3). Generally speaking, the major reasons identified for children’s growth failure can be subsumed under two general rubrics, namely, organic reasons such as acute and chronic diseases (infection) interfering with the absorption or metabolism of nutrients, and inorganic reasons such as not receiving enough food, decreased appetite, mothers’ insufficient knowledge regarding correct child feeding ways, and parental employment status (4).

Not paying enough attention to the delay or stop in children’s growth, which is common and prevalent and typically occurs in children below 5 yr of age, can lead to further major complications such as an increase in mortality, a rise in other related diseases, a decrease in learning rate, and physical, mental, and emotional disorders (5).

One of the ways adopted to achieve this aim and to monitor growth disorders in children is periodic and regular monitoring of children’s height and weight and screening disorders in them (6,7). For example, growth curve is a simple and easy way for monitoring children’s sanitary and nutritional status, which can simply be used by health care professionals (7, 8).

“In monitoring children’s growth and applying growth curve, the trend and direction of the curve are of paramount importance such that between 3 and 97 percentile (as a normal range) four different conditions can be identified” (9). As for the first condition, the trend and direction of growth are upward and parallel with the universal standards construed as being normal. In the second condition, likewise the first condition, the growth curve trend is upward, however with a lower slope which is indicative of growth slowdown. In the third condition, the growth curve trend is horizontal suggesting growth cessation. Finally, the trend of growth curve is downward indicating growth failure. From among the above-mentioned conditions, the second to fourth conditions are conceived of as being abnormal and require urgent treatment (7, 8).

Growth failure is more prevalent in developing countries and in these countries; children’s physical growth is less than international standards. 9.5% of the children below 5 yr of age were moderately and severely underweight in Iran. Moreover, the prevalence of stunting and wasting was 13.9 and 5.3, respectively (10).

From a statistical perspective, likewise, most of the similar studies carried out to examine growth failure in children below 5 yr of age, mostly deployed descriptive statistics or no specific statistical modeling was done on the data (11–13).

In monitoring growth failure in the growth curve of the children below 2 yr of age, children might experience repetitive or recurrent growth failures (14). For the modeling of the correlated data in survival analysis, diverse methods have been proposed and applied. From among the proposed methods, methods based on events’ rate, gap times between events and complete intensity functions are the most common methods. Methods based on events’ rate are used firstly when the repeatability of recurrent events is high and secondly when the incidence of a recurrent event does not have a significant effect on the incidence of the next event. These methods are mostly based on counting process and specifically, on poison process. Besides, in these methods, the number of events in non-overlapping time intervals are independent and identically distributed (15).

Given the recurrent nature of the incidence of growth failure in the growth curve of the children below 2 yr of age, models dubbed recurrent events models are useful for the investigation of growth failure and effective factors on it.

Finally, given the importance of growth failure, its adverse effects and serious complications on family and community health, the gap in the use of statistical models (specifically, the use of recurrent events model) in the analysis of children growth failures, and lack of similar studies on the population of the western part of the country, the present study, using a proportional rate model, made an attempt to investigate the effect of some of the effective factors on growth failure in the height and weight of the children of Khorramabad, central Iran below 2 yr of age.

## Materials and Methods

In this longitudinal retrospective study, all bellow 2 yr old children born in Khorramabad, during 2009 to 2011 were at the study time (from Aug to Sep 2014), constitute the study population. Moreover, the study population had a record in one of urban health centers, visited the health center for vaccination or for monitoring their growth status, had at least 37 wk of gestational age at birth time and finally, did not suffer from any congenital disease (according to the information in their household records).

To ensure the confidentiality of the information of the household records, all the data gathering and analysis procedures were conducted anonymously.

Multistage sampling was used such that firstly, the strata were selected and consequently, in each stratum, one-stage cluster sampling was used. To put it more precisely, at first, Khorramabad urban health centers were divided to fall into three major strata namely northern, central and southern urban health centers. In the next stage, in each stratum, a number of health centers were selected (proportional to size) to constitute the clusters. Using systematic random sampling, two health centers from the north, two health centers from the south and three health centers from the central part of the city were selected as clusters. Finally, referring to the household records in the selected health centers, a sample of 2013 children born during the successive years of 2009 to 2011 was entered into the study.

In the next stage, after conducting the sampling procedures, the required information of the household record was inserted into a self-designed data gathering form and its face validity and content validity were approved based on the literature review and the comments of four faculty members of Lorestan University of Medical Sciences. This form includes variables such as: times of child’s visit to the health center, child’s gender, birth order, parents’ educational level, parents’ occupation, family size, exclusive breast-feeding during the first 6 month of child’s birth and child’s height and weight growth status compared to the previous visit to the health center.

To record children’s weight status during all ages, a binary coding method was adopted (normal growth: upward sloping growth curve that is parallel to the standard curve, growth failure: upward growth curve with a lower slope, horizontal or downward growth curve). Moreover, to record children’s height growth status during all ages, a similar two-mode coding method was deployed (normal growth: upward sloping growth curve that is parallel to the standard curve, growth failure: upward growth curve with a lower slope and horizontal growth curve). To ensure and maintain the reliability of observations, each growth curve was examined by two public health experts separately and if there was not an agreement between them as to growth failure for a given child, the record of that child was not taken into account in the following investigations.

Children’s gender, birth order, parents’ educational level, parents’ occupation, family size, exclusive breastfeeding during the first 6 months of child’s birth comprised the primary independent variables of the present study. However, to avoid collinearity, the three variables of father’s educational level, mothers’ occupation, and family size were not taken into account for the modeling of the data. The dependent variable of the present study, on the other hand, was “the time to incidence of growth failure in height and weight”. In each height and weight monitoring, two situations might occur: observing growth failure (the occurrence of the event) or observing no growth failure (right censoring). Given that growth failure is recurrent, thus, the model adopted for the data analysis should be able to consider this feature.

To describe data, frequency tables, and to model the data, a recurrent events model construed as a proportional rate model, was deployed. Kleinbaum and Klein referred to this type of recurrent event model as “Counting Process approach” (16).

All the data were described and analyzed using SAS software, ver. 9.2 via PROC PHREG at the significance level of 0.05.

## Results

Male children constituted 52.2% of the population (1051 persons) and 50.8% of the children under investigation (1022 persons) were second or higher-order, in terms of birth order. Furthermore, about 59.1% of mothers (1189 persons) and 61.7% of fathers (1242 persons) had either a high-school diploma or higher educational levels. From among the parents under study in the present research, 87.8% of mothers were householders (1767 persons) and 80.9% of the fathers (1628 persons) were either employed or self-employed. As for family size, 75.8% of the households (1351 cases) had 3 to 4 members. Finally, 86.1% of the children (1734 persons) were exclusively breastfed during the first six months of their life. Table 1 describes the demographic and background information of the children under the study.

**Table 1:** frequency distribution of the children below two years of Khorramabad, Iran in 2014, based on background and demographic characteristics

***Variable***	***Category***	***Frequency***	***Percent***
Child’s gender	Female	962	47.8
	Male	1051	52.2
Child’s birth order	1st	977	49.2
	2nd or higher	1022	50.8
Mother’s educational level	Less than high school diploma	821	40.9
	High school diploma or higher	1189	59.1
Father’s educational level	Less than high school diploma	767	38.3
	High school diploma or higher	1242	61.7
Mother’s occupation	Housewife	1767	87.8
	Employed	243	12.2
Father’s occupation	Worker/unemployed	377	19.1
	Employee/self employed	1628	80.9
Family size	3–4	1531	75.8
	≥ 5	475	24.2
Exclusive breastfeeding	No	218	13.9
	Yes	1734	86.1

The number of the incidences of growth failure in the height and weight growth curves of the children under two years of age varied between 0–7 times. The frequency distribution Table 2 shows the distribution of the number of growth failures in the height and weight growth curves of the children less than two years of age.

**Table 2:** Frequency distribution of the children below two years of Khorramabad, Iran in 2014, based on the number of incidence of growth failure in weight and length

***Variable***	***Category***	***Frequency***	***Percent***
Number of incidence of growth failure in weight	0–1 times	361	18.1
	2 times	385	19.2
	3 times	698	34.9
	4 times	381	19.0
	≥5 times	176	8.8
Number of incidence of growth failure in height	0–1 times	99	5.0
	2 times	409	20.4
	3 times	779	38.9
	4 times	576	28.8
	≥5 times	138	6.9

Using recurrent events model to determine effective factors on the growth failure in height and weight of the children under investigation, the effect of fathers’ occupation on children’s weight growth failure was significant (*P*<0.001). The rate of growth failure in the weight of children whose father was either worker or unemployed was about 12.2%, more than children whose parents were employee or self-employed (95% CI: 6.7% - 18.2%). Moreover, the effect of mothers’ educational level on growth failure in children’s weight was significant (*P*=0.049) such that rate of growth failure in the weight of children whose mothers’ educational level was high-school diploma or higher is 4.7% more than children whose parents had lower educational level (95% CI: 0.6%–9.0%). However, the effect of gender on growth failure in children’s weight was not significant (*P*=0.080). On the other hand, the rate ratio of growth failure in the weight of male children was 3.7% more than their female counterpart. In the present study, the effect of the two variables of birth order and exclusive breastfeeding on growth failure in children’s weight is significant (*P*=0.877, *P*=0.684) (Table 3).

**Table 3:** Estimated RR for the rate of growth failure in the weight of the children below two years in the city of Khorramabad, Iran in 2014, using recurrent events model

***Variable***	***Category***	***Estimated rr***	***95% ci***	***P-value***
Child’s gender	Female	Reference		
	Male	1.037	0.988–1.076	0.080
Child’s birth order	1st	Reference		
	2nd or higher	1.011	0.963–1.060	0.684
Mother’s educational level	Less than high school diploma	Reference		
	High school diploma or higher	1.047	1.006–1.090	0.049
Father’s occupation	Employee/self employed	Reference		
	Worker/unemployed	1.122	1.067–1.182	< 0.001
Exclusive breastfeeding	No	Reference		
	Yes	1.005	0.945–1.067	0.877

The results of modeling effective factors on growth failure in children’s length, obtained using recurrent events model, indicate that the effect of mothers’ educational level on growth failure in children’s length is significant (*P*=0.046). The rate ratio of growth failure in the length of children whose mothers’ educational level is high school diploma or higher is averagely 4.2% more than children whose mothers had lower levels of educational (95%, CI: 2.3%–7.5%). The effect of exclusive breastfeeding on the growth failure in the length of children was not significant (*P*=0.098). However, the rate ratio of growth failure in the length of children exclusively breastfed was 3.9% more than those children not exclusively breast-fed. In the present study, the effect of children’s gender, fathers’ occupation, and birth order on the growth failure in children’s length was shown to be non-significant (*P*=0.831, *P*=0.510, *P*=0.202) (Table 4).

**Table 4:** Estimated RR for the rate of growth failure in the length of the children below two years in the city of Khorramabad, Iran in 2014, using recurrent events model

***Variable***	***Category***	***Estimated RR***	***95% CI***	***P-value***
Child’s gender	Female	Reference		
	Male	1.018	0.993–1.044	0.202
Child’s birth order	1st	Reference		
	2nd or higher	0.997	0.971–1.023	0.831
Mother’s educational level	Less than high school diploma	Reference		
	High school diploma or higher	1.042	0.925–0.977	0.046
Father’s occupation	Employee/self employed	Reference		
	Worker/unemployed	0.988	0.958–1.020	0.510
Exclusive breastfeeding	No	Reference		
	Yes	1.039	0.986–1.084	0.098

## Discussion

The present study aimed to investigate some effective factors on the growth failure in the weight and length of the children aging below two years, revealed that the two variables of fathers’ occupation and mothers’ educational level have a significant effect and child’s gender has a non-significant (but considerable) effect on the growth failure in the weight of the children. However, the effect of birth order and exclusive breastfeeding in the first six months after birth on the growth failure in children’s weight is not significant. Besides, mothers’ educational level has a significant effect on the growth failure in the length of children and exclusive breastfeeding in the first six months after birth have a non-significant (but considerable) effect on the growth failure in the length of children.

However, the effect of variables such as fathers’ occupation and birth order on growth failure in the length of children is not significant. Moreover, in the present study, quite contrary to the similar and previously conducted studies in which children’s age and weight are used directly, “the time of the incidence of recurrent growth failures in children’s weight and length” is used. Thus, the differences between the results of the present study and other studies in the literature can be attributed to and accounted for by the abovementioned difference in the choice of dependent variable, because of special nature of dependent variables in survival analysis

In the present study, the effect of gender on the rate of growth failure in children’s weight and length was revealed non-significant. However, in a rather similar research, on the children below 2 yr of age of urban areas, except at one month of age, the average weight of male children is higher than their female counterpart. Furthermore, before the age of two months and at the age of two, there was not a significant difference between the weight of male and female children (11). From birth to three years, there was not a significant difference between the average length of male and female children. However, during all ages, the average weight of male children was reported to be higher than their female counterpart (3). The most important reason invoked to account for the above-mentioned inconsistency is that although weight and length growth curve of male children is higher than the weight and length growth curve of their female counterpart, in all ages, the two curves are almost parallel. Accordingly, no significant difference is observed in the slope or the trend of height and weight growth curve.

In the present study, weaning was not a contributing factor in growth failure in the length and, most specifically, in the weight of the children below two years of age. Although before six months of age, the rate of growth failure in the weight of children breastfed was comparatively less than weaned children, after six months and after weaning, the rate of growth failure in the weight of children breastfed was higher, compared to other children. Accordingly, the main effect of exclusive breastfeeding on growth failure in the weight of children was non-significant. In a study conducted on children below the age of two, weaning was one of the decisive factors in growth failure in children’s weight and if a proper nutrition was not substituted for it, or if the child was not provided with complementary food in the due time, it would have a considerable effect on growth failure in children’s weight led to weight loss (17,18).

Weaning causes children to be deprived of a food with healthful, nutritional, immunologic, developmental, psychological and social benefits for a long or short period of time (19–21). The effects of weaning and starting complementary food on growth failure were significant (17, 22, 23).

As for the effect of parents’ educational level on growth failure in the length and weight of children, as mothers’ educational level increases, weight loss in children below two years of age decreased (24). In line with the results of the present study, there was a relationship between mothers’ educational level and children’s weight loss such that, as mothers’ education increased, weight loss decreased. In the same line of research, the decrease in children’s weight loss was portrayed as mothers’ educational level increases (6, 25). However, the results of the abovementioned studies are not in line with present study. Mothers’ educational level did not have a significant effect on growth failure, it reduced growth failure, in general (17, 18). In the present study, a higher percentage of mothers with higher education were employed, compared to less educated mothers (18.5% vs. 2.8%). Thus, because of part-time presence at home, employed mothers have a poor performance regarding child’s feeding and have a rather negative attitude towards exclusive breastfeeding during the first six months of age.

In the present study, the effect of fathers’ occupation, as an indicator of family’s socioeconomic status, was highlighted such that children from wealthier families (self-employed and employees) had a better growth trend, compared to children of other families (workers and unemployed persons). Mothers’ employment status, rather than fathers’ occupation, was taken into account and its effect on growth failure was investigated. For example, the rate of growth failure in children, whose mother’s employment was higher, compared to children whose mothers were homemaker (26).

In a study conducted on the children of Tehran below the age of 5 yr, the only effective factor on children’s weight was children’s birth order (7), while, in the present study, the effect of this variable was non-significant. However, the rate of growth failure in the weight of children with second or higher birth order was higher, compared to the first-order born children.

The strength of this study is the use of large sample size and the use of recurrent events model in the analysis of the data in that this model can take the correlation between different times of the incidence of growth failure into account and therefore can raise the accuracy of the statistical estimations comparing to traditional models. The most important limitation of the present study was that information of the household records was not complete and the effect of some of the confounding variable could not be controlled. Moreover, only urban children were opted for and drawn upon as the population; accordingly, the results cannot be generalized to rural children. From a statistical perspective, it is suggested to use a time-dependent rate model for the analysis of the data (26).

## Conclusion

Raising mothers’ awareness in low-income families along with changing the attitude of working and educated mothers regarding the correct principles of feeding children below two years of age can be conceived of as the most effective ways of coping with and reducing growth failure in length and weight. Moreover, given the high rate of growth failure in the children of Khorramabad aging below two years, designing and implementing regional interventions to improve the growth and nutrition of these children appear to be necessary.

## Ethical considerations

Ethical issues (Including plagiarism, informed consent, misconduct, data fabrication and/or falsification, double publication and/or submission, redundancy, etc.) have been completely observed by the authors.
